# Wavelet Scattering Features for Robust Subject-Independent EMG-Based Speech Recognition: A Comparative Analysis

**DOI:** 10.3390/s26206440

**Published:** 2026-10-12

**Authors:** Jumana Matouq, Omar Abulayla, Rami N. Khushaba, Jamal I. Al-Nabulsi, Adel Al-Jumaily

**Affiliations:** 1 Department of Biomedical Engineering, School of Applied Medical Sciences, German Jordanian University, Amman 11180, Jordan; o.abulayla@gju.edu.jo; 2 Transport for NSW, Alexandria, NSW 2015, Australia; rami.khushaba@transport.nsw.gov.au; 3 Medical Engineering Department, Faculty of Engineering, Al-Ahliyya Amman University, Amman 19111, Jordan; 4 School of IT and Engineering, Melbourne Institute of Technology, Sydney, NSW 2000, Australia; aal-jumaily@mit.edu.au; 5 Department of Computer Science and Software Engineering, University of Western Australia, Crawley, WA 6009, Australia; 6 School of Information Technology, UNITAR International University, Petaling Jaya 47301, Selangor Darul Ehsan, Malaysia; 7 School of Science, Edith Cowan University, Joondalup, WA 6027, Australia

**Keywords:** electromyography (EMG), feature representation, Mel-Frequency Spectral Coefficients (MFSC), speaker dependence, wavelet scattering transform (WST), SDG 3

## Abstract

**Highlights:**

**What are the main findings?**

- The WST-based pipeline improved subject-independent EMG speech recognition across four temporal architectures.
- A Transformer backend with the WST-based pipeline achieved 85.82% accuracy on 20 unseen speakers.

**What are the implications of the main findings?**

- Variance analysis suggests that features from the WST-based pipeline carry less speaker-specific information than MFSC features.
- The WST-based pipeline provides a promising foundation for subject-independent EMG-based AI systems.

**Abstract:**

Electromyography (EMG)-based speech recognition provides a non-acoustic alternative in high-noise environments and serves as a prospective foundation for assistive technologies for users with speech impairments. While prior work has largely focused on model architecture, the impact of EMG feature representations on the robustness and subject-independent generalization of deep learning models remains underexplored. This study aims to investigate how feature representation influences subject-independent EMG speech recognition by comparing two feature extraction pipelines—a conventional Mel-Frequency Spectral Coefficients (MFSC) pipeline and a Wavelet Scattering Transform (WST)-based pipeline—across four temporal back ends: gated recurrent unit (GRU), Transformer, ReMamba, and BiMamba. Recordings from 100 participants during voiced (vocalized) speech reading a closed set of fixed sentences were used, of which 20 participants’ recordings were excluded from model development and used exclusively for subject-independent testing to verify generalization. The speaker dependence of both representations was additionally examined with a participant-level variance analysis. The WST-based pipeline achieved higher sentence-level accuracy than the MFSC pipeline with every backend, with the best result reaching 85.82% on unseen participants. For the Transformer backend, accuracy increased from 78.59% with MFSC to 85.82% with the WST pipeline. Speaker identity explained 12% of the feature variance in the WST pipeline compared with 69% for MFSC. When the filtering pipeline used for MFSC was applied before scattering extraction, accuracy decreased by 2.16 percentage points, indicating that this preprocessing may suppress EMG information useful to the model. Collectively, the findings identify the WST-based pipeline as a less speaker-dependent EMG feature representation that improves cross-subject performance across different sequence models and may provide a basis for intelligent EMG speech-recognition systems.

## 1. Introduction

Speech is a primary channel through which humans convey knowledge, emotion, and intent. Its role now extends beyond interpersonal communication to human–computer interaction, where it supports technologies ranging from virtual assistants to accessibility tools [1].

Audio-based automatic speech recognition (ASR) is highly successful under ordinary conditions, but recognition can deteriorate substantially when acoustic interference is strong. This limitation has accelerated the development of non-acoustic interfaces for vocalized speech recognition (VSR) and Silent Speech Recognition (SSR) [2,3], utilizing signals like visual cues (lip-reading) [4], ultrasound [5], radar [6], and biosignals, such as electroencephalography (EEG) and electromyography (EMG) [7,8,9]. A recent attention-based VSR approach has further demonstrated improved recognition performance and generalization to unseen speakers in noisy conditions [10]. Among biosignals, surface EMG is a non-invasive modality used to assess muscle activity and has been studied in rehabilitation, health monitoring, and wearable sensing applications [11]. Unlike VSR, SSR aims to ensure accurate communication in situations where audible speech is either unavailable or undesirable. In such circumstances, EMG signals offer an articulation-based approach that bridges neuromuscular activity and speech production. While surface EMG has been investigated across both VSR and SSR settings, the present benchmark explicitly evaluates voiced (vocalized) speech. Demonstrating robust cross-subject feature representations under voiced conditions provides an essential precursor before extending these representations to unvoiced or silent speech interfaces.

Previous research using EMG in SSR and VSR showed several benchmarks in isolated word recognition and promising progress toward sentence-level EMG-based speech recognition using deep learning approaches, e.g., 90% (using EMG Spectrogram + bidirectional long short-term memory (Bi-LSTM)) [12], 12.2% word error rate (WER) (MONA LISA, Cross-modal + large language model (LLM) scoring) [13], 90.33% for EMG and 98.67% for EEG + EMG [14]. In a silent-text classification setting, Kang et al. [15] achieved 95.63% accuracy and 95.62% F1-score for eight frequently used English letters using fused EMG and piezoelectric sensor signals. On the other hand, recognizing full sentences is more difficult, yet recent state-of-the-art progress has been made, with results showing some variation, e.g., 90.76% (EMG-based, 100 Mandarin Phrases, 28 subjects, random 80/20 split of pooled data) [16], 75.53% (EMG-based, 100 Mandarin Phrases + Silence, 70-subject training set and 20-subject testing set) [2] and 93.33% (EMG-based sentence recognition task, 1 subject) [14]. Xie et al. [17] further showed that using data augmentation techniques can greatly lower error rates in SSR when translating silent facial EMG signals into text. Salomons et al. [18] further demonstrated that electrode type, placement, and targeted facial and neck muscles are important considerations in the design of EMG-based silent speech interfaces. Nevertheless, achieving robust subject-independent sentence recognition remains a significant challenge because of the high variability and non-stationary characteristics of EMG signals.

Baseline experiments on the audio, visual, and Electromyographic (AVE) speech dataset showed that conventional frequency-domain EMG representations, i.e., log Mel-Frequency Spectral Coefficients (MFSC), provide a strong foundation for sentence-level EMG-based speech recognition. This observation agrees with an earlier comparison of short-time Fourier transform (STFT), MFSC, and Mel-Frequency Cepstral Coefficients (MFCC), in which MFSC produced the strongest EMG-based SSR results across several deep networks [16], while MFCC-based feature extraction has also been applied to detection of manipulated audio content [19]. Mel-frequency and wavelet-based features have also shown strong classification performance in pathological voice analysis [20]. In addition, Moslhi et al. [21] showed that the choice of feature extraction method can significantly influence surface electromyography (sEMG) classification performance, comparing Fourier- and wavelet-based representations across multiple public EMG datasets. Nevertheless, it remains unclear how much the selected representation determines model robustness and transfer to participants not encountered during training. This gap motivates the study of alternative signal descriptions that offer greater stability while retaining discriminative content, especially for one-dimensional modalities such as EMG and audio [22,23]. Input design is also becoming increasingly important as the field moves away from high-density EEG/EMG arrays toward wearable systems with fewer channels. Under these practical constraints, an informative and disturbance-tolerant representation is particularly valuable [24].

Despite recent progress, physiological differences, changes in electrode location [24], and variation between users continue to impede reliable EMG speech recognition. These sources of variability motivate both stronger signal representations and effective temporal models. Thus, the aim of this study is to systematically investigate the effect of EMG feature representation on subject-independent speech recognition by comparing MFSC and the WST across four temporal backend architectures—GRU, Transformer, ReMamba, and BiMamba—using a closed-set classification task with previously unseen test speakers. To address this aim, we use the recently released AVE Speech corpus [2] to determine how the EMG representation affects robustness and generalization to unseen participants. AVE Speech is a large Mandarin sentence-recognition benchmark comprising synchronized audio, video of the lip region, and six EMG channels for 101 classes. The present analysis deliberately uses only EMG so that the contribution of this modality can be examined without the confounding benefit of multimodal fusion. Using the same temporal backends, the conventional log-MFSC pipeline is compared with a WST-based pipeline. Because scattering features retain discriminative structure while remaining stable under small signal deformations, they are well suited to the non-stationary dynamics of speech-related biosignals. We test the hypothesis that their multiscale description of temporal activity improves robustness and cross-subject recognition. The following sections define both frontends, benchmark them with several temporal backends, and discuss the resulting accuracy-efficiency trade-offs and directions for further research.

## 2. Materials and Methods

**Figure 1:**
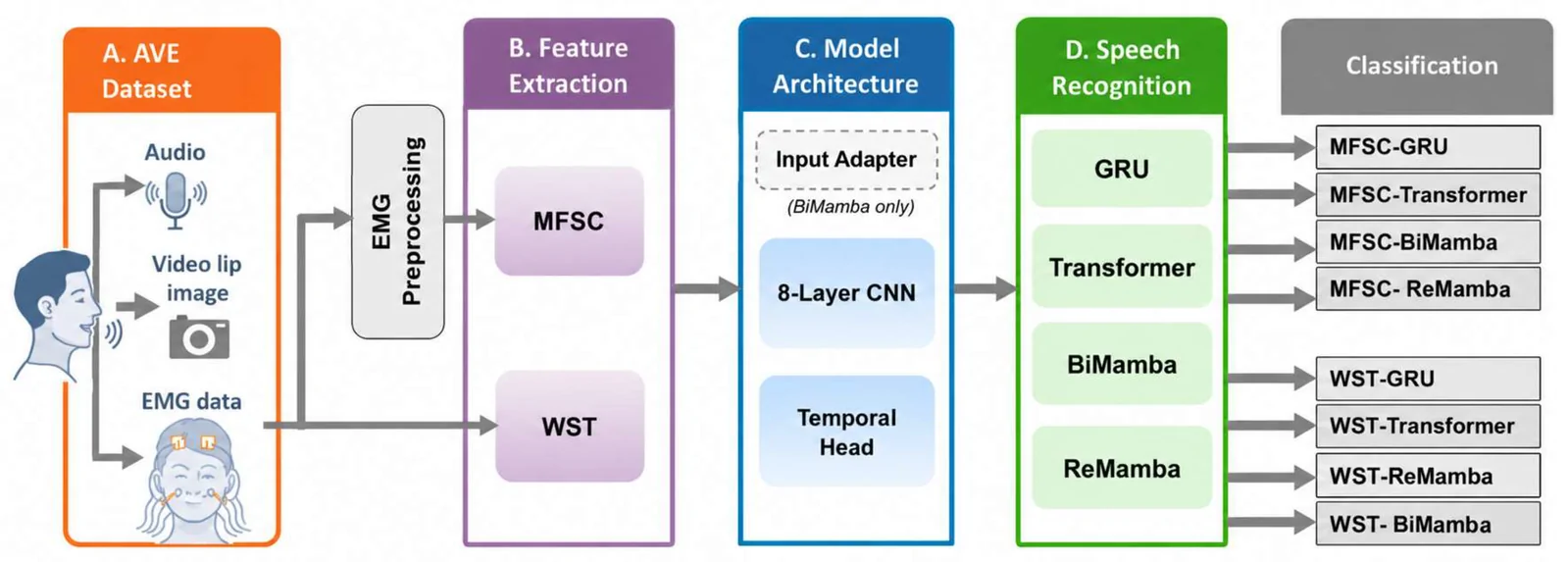
Illustration of the electromyography (EMG)-based speech recognition pipeline, using the AVE Speech dataset [2].

Figure 1 provides an overview of the entire pipeline, illustrating the AVE dataset, pre-processed EMG signals, feature extraction (MFSC and WST), model architecture, speech recognition, and classification. Each component of this pipeline is explained in detail as follows.

### 2.1. AVE Dataset

**Figure 2:**
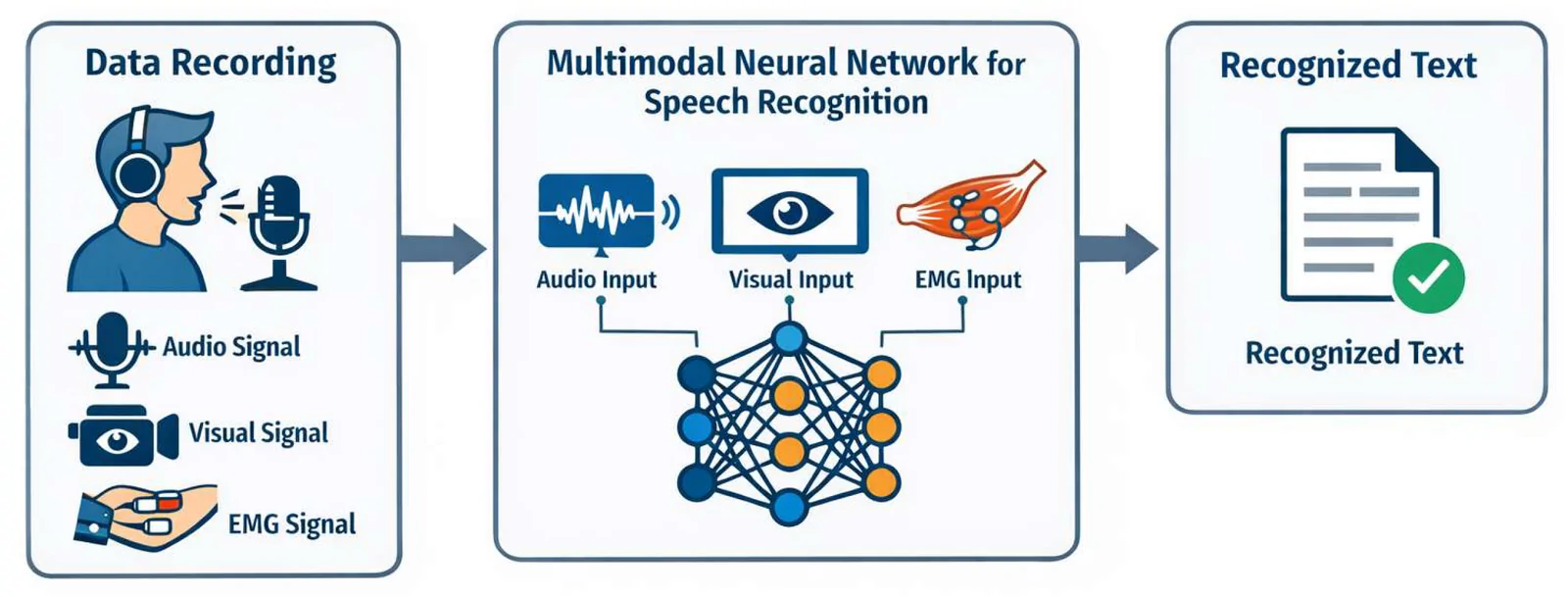
Illustration of the multi-modal speech recognition pipeline using audio, visual, and EMG signals in the AVE dataset.

The openly available AVE corpus contains synchronized audio, lip-region video, and six-channel surface EMG from 100 native Mandarin speakers. Each participant repeated 100 sentences selected from every day and medical-need scenarios ten times, providing more than 55 h of recordings for each modality [2]. The multimodal acquisition setup is illustrated in Figure 2, with the present experiments using only facial and neck EMG. EMG was sampled at 1 kHz from electrodes positioned over facial and neck muscles involved in articulation, including the mentalis, risorius, levator anguli oris, digastric, mylohyoid, and platysma. To our knowledge, AVE Speech is the first public resource to combine sentence-level EMG with recordings from such a large participant cohort. It consequently enables direct comparison of EMG frontends, including MFSC and WST, while supporting rigorous assessment of between-subject variation and cross-speaker generalization in voiced EMG sentence recognition. Our experiments therefore use the EMG modality alone and adopt a participant-disjoint split: subjects 1–70 form the training set, subjects 71–80 the validation set, and subjects 81–100 the test set. The task is explicitly formulated as closed-set classification across 101 fixed sentence classes (100 daily/medical Mandarin sentences plus one blank class). All surface EMG recordings in the AVE dataset were collected during voiced (vocalized) speech. While our evaluation assesses cross-subject generalization on 20 unseen test subjects, the sentence classes in the test set are identical to those used during training. Consequently, generalization performance reflects transfer to unseen speakers under fixed sentence conditions and does not imply generalization to unseen text or open-vocabulary speech. The corpus can be accessed at https://huggingface.co/datasets/MML-Group/AVE-Speech (accessed on 15 October 2025).

### 2.2. Feature Extraction

This section describes the two EMG frontend representations evaluated in the experiments: MFSC and WST.

#### 2.2.1. MFSC

MFSC expresses each EMG channel in the time–frequency domain by measuring short-window signal energy through a bank of filters spaced on the mel scale. The resulting two-dimensional array is compact while retaining the evolution of spectral content over time. Wu et al. [16] previously found this representation effective for EMG speech recognition, since articulation produces organized, time-varying spectral structure that can be captured by spectrogram-based features.

To maintain comparability with the AVE Speech baseline, we reproduced the MFSC extraction and EMG preprocessing procedure of Zhou et al. [2] and used the same settings for each backend. Four second-order IIR notch filters first attenuated interference at 50, 150, 250, and 350 Hz. A fourth-order Butterworth filter then retained frequencies between 10 and 400 Hz. At the original 1 kHz sampling rate, the short-time Fourier transform used a 200-sample analysis window and a 50-sample hop. The spectrum was projected through 36 mel filters and log-compressed, yielding 36 output frames per channel.

In our pipeline, the MFSC features are precomputed and stored before training. This guarantees that all models operate on fixed-length, consistently normalized inputs, reducing sample-to-sample variability and improving training stability. These features are computed independently for each EMG channel to obtain a consistent time–frequency representation. A 1.75 s segment was used, yielding exactly 36 frames, so no padding or truncation is required. Finally, each 6 × 36 × 36 array is normalized by its own mean and standard deviation. After these operations, one channel yields a 36 × 36 log-mel time–frequency array. Arrays from all six channels are then combined into an input tensor with dimensions (1, *C*, 36, 36), where *C* = 6.

#### 2.2.2. WST

**Figure 3:**
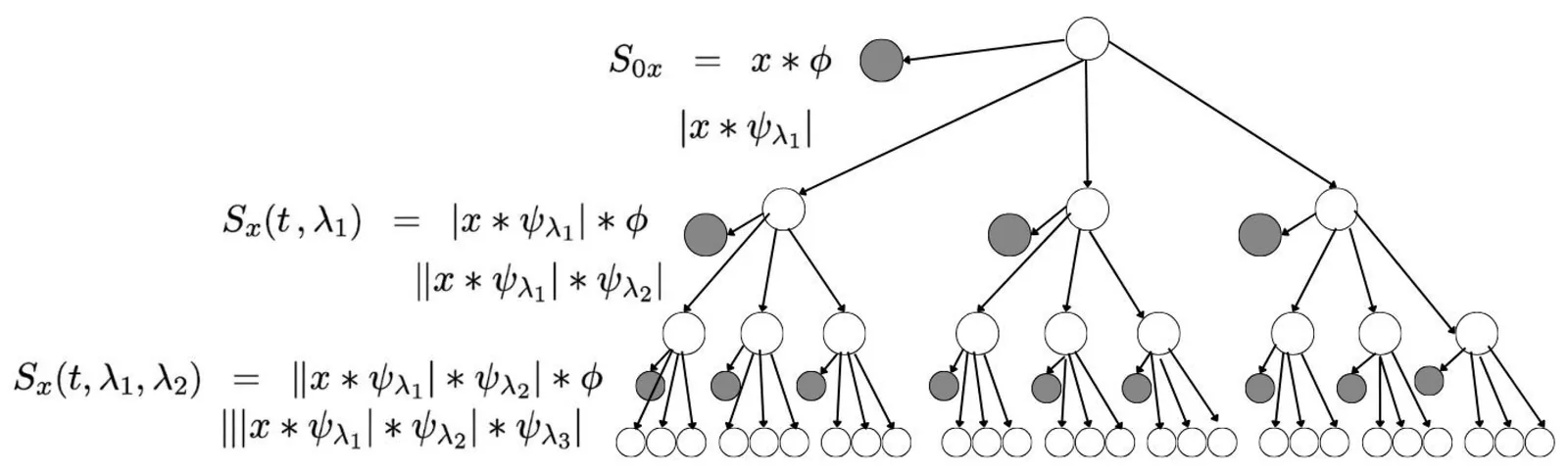
Illustration of the wavelet scattering transform based on ref. [25].

WST forms a hierarchical signal description through repeated complex-wavelet convolution, modulus operations, and low-pass averaging, while providing invariance to translation [25]. In contrast to STFT and MFSC, which apply predetermined spectral mappings, scattering employs a structured bank of fixed wavelets whose mathematical construction provides stability to small temporal deformations [25,26]. Successive scattering layers, depicted in Figure 3, represent increasingly complex modulation patterns. These characteristics are relevant to EMG, whose observations are non-stationary and can change with electrode placement or repeated articulations. The scattering coefficients are defined below.

Based on [25,26], for an EMG waveform *x*(*t*), the zeroth-order scattering coefficient *S*_0_*x*(*t*) is obtained by convolving the input signal with a low-pass averaging filter *ϕ*(*t*), capturing its low-frequency content:

*S*_0_*x*(*t*) = *x*(*t*) ∗ *ϕ*(*t*), (1)

The first-order scattering coefficients *U*_1_*x*(*t*,*λ*_1_) encode the allocation of signal content among frequency bands. For each complex wavelet $\psi_{\lambda_{1}}(t)$ in the selected family, convolution is followed by the modulus operation:

(2) $$U_{1}x(t,\lambda_{1})=|x(t)*\psi_{\lambda_{1}}(t)|.$$

The result describes localized, instantaneous energy within each subband. Local averaging is subsequently applied to make the representation insensitive to small displacements in time.

*S*_1_*x*(*t*,*λ*_1_) = *U*_1_*x*(*t*,*λ*_1_) ∗ *ϕ*(*t*). (3)

The resulting representation encodes how EMG energy is distributed across multiple frequency scales and is closely related to the dominant muscle activation patterns associated with phoneme and syllable production.

For mathematical completeness, we continue the description of WST below to account for the subsequent orders of scattering coefficients, though different software libraries may offer varying capabilities in terms of generating these subsequent coefficients. The second-order scattering coefficients *U*_2_*x*(*t*,*λ*_1_,*λ*_2_) show the temporal variations in the first-order amplitudes. For each first-order path λ_1_, the modulus-transformed wavelet response *U*_1_*x* is filtered again with another wavelet $\psi_{\lambda_{2}}(t)$:

(4) $$U_{2}x(t,\lambda_{1},\lambda_{2})=|U_{1}x(t,\lambda_{1})*\psi_{\lambda_{2}}(t)|,$$

averaging these responses yields

*S*_2_*x*(*t*,*λ*_1_,*λ*_2_) = *U*_2_*x*(*t*,*λ*_1_,*λ*_2_) ∗ *ϕ*(*t*). (5)

These second-order terms characterize changes in subband energy over time, including rapid articulatory transitions, coarticulation, contraction-relaxation patterns, and phoneme boundaries. Rather than being manually constructed as in delta-MFCC features, these modulations arise from the scattering cascade and retain an explicit multiscale interpretation.

Higher-order coefficients are obtained by repeating this procedure. For example, the third-order coefficients are defined as

(6) $$U_{3}x\left(t,\lambda_{1},\lambda_{2},\lambda_{3}\right)=|U_{2}x\left(t,\lambda_{1},\lambda_{2}\right)*\psi_{\lambda_{3}}\left(t\right)|,$$

with the corresponding averaged form

*S*_3_*x*(*t*,*λ*_1_,*λ*_2_,*λ*_3_) = *U*_3_*x*(*t*,*λ*_1_,*λ*_2_,*λ*_3_) ∗ *ϕ*(*t*). (7)

Higher scattering orders generally have smaller magnitudes and are often omitted to control computational costs. Nevertheless, third-order coefficients can describe fine co-modulations that help separate closely related articulatory states in speech EMG. Taken together, the scattering representation offers four relevant properties: insensitivity to translation and stability under modest temporal deformation; coverage of EMG dynamics from slow envelopes to rapid modulations; coefficients that can be related to muscle-activation behavior; and fixed filter banks that do not need to be estimated from a limited training corpus. These attributes may reduce estimation variance and improve generalization in EMG speech recognition, where noise, session-to-session changes, electrode displacement, and variation in articulatory effort are unavoidable. The value of WST has likewise been demonstrated in several other signal-analysis settings [25,27,28,29].

In the current implementation, the same 1.75 s segment is used for both MFSC and WST and each channel is scaled by its root-mean-square (RMS) value. The scattering transform is computed with Kymatio (version 0.3.0) with parameters of Scattering 1D with Morlet wavelets, *J* = 4, *Q* = 4, max_order = 3, averaging scale of 16 samples. In this implementation of Kymatio, the transform strictly generates zeroth-order (*S*_0_) and first-order (*S*_1_) scattering coefficients (based on the design of Kymatio library itself). Given the RMS scaling in our WST-based pipeline, the zeroth-order coefficient (*S*_0_) was explicitly discarded via feature slicing when constructing the feature tensor fed to the subsequent stages of the pipeline, hence relying exclusively on the first-order scattering coefficients (*S*_1_). This in turn provides multi-rate temporal energy envelopes across subbands while maintaining a compact feature representation. These coefficients are log-compressed, normalized per channel over the recordings of the same session, and extended with their first and second temporal differences (Δ, Δ^2^). A fixed, untrained 1 × 1 channel projection, bilinear resampling of the coefficient axis to 36 rows, and pairwise frame averaging then give an input of a 6 × 36 × 53 feature tensor. The 36-feature dimension is explicitly imposed by interpolation to match the number of features in MFSC (the number of Mel bands), while 53 represents the temporally downsampled scattering positions. Hence, both the MFSC and WST representations contained 36 coefficients per EMG channel but differed in their native temporal resolution (36 and 53 temporal positions for MFSC and WST, respectively). These temporal dimensions were retained to avoid altering the intrinsic time–frequency characteristics of either representation through artificial dimensional matching, which could itself introduce an additional experimental bias. The resulting feature tensors (6 × 36 × 36 for MFSC and 6 × 36 × 53 for WST) were then fed to the subsequent recognition backends.

Overall, the two pipelines, i.e., MFSC and WST, while having the same number of features, are different in several aspects extending beyond the transform itself. Filtering is applied only before MFSC, whereas RMS scaling, session-level normalization, the Δ/Δ^2^ features, channel projection, and interpolation are used only in WST. The WST input also has more frames (53 versus 36; 11,448 versus 7776 values), although both contain 216 values per frame. The comparison is therefore between two complete feature pipelines rather than between the transforms alone.

### 2.3. Model Architecture

As shown in Figure 1, the proposed framework operates on 6-channel EMG signals using two EMG representations: MFSC and WST. For each backend, the same network was used for both representations, except for minor differences in the pooling layers of the ReMamba backend. The model starts with a shared convolutional frontend, using the eight 3 × 3 convolutional layers as in the original AVE architecture [2], followed by Batch Normalization, Rectified Linear Unit (ReLU) activation, max-pooling, and dropout.

A small input adapter is used only in the BiMamba models, and it is applied to both MFSC and WST inputs. It consists of per-channel Batch Normalization and a depth-wise 1 × 3 convolution (30 trainable parameters, as included in Table 1). The GRU, Transformer, and ReMamba models do not use an adapter, and the untrained channel projection of the WST pipeline is not counted as a model parameter. After the convolutional layers, the resulting feature map is flattened into a tensor of shape (B, T, 256), where B is the batch size, T represents the number of temporal tokens produced by the CNN frontend, and 256 is the feature dimension per token. The resulting feature sequence is then processed by a temporal modeling backend. Because of the pooling, the resulting token sequences are short (one or two tokens for the GRU and Transformer models and up to 53 for BiMamba).

### 2.4. Speech Recognition Models

#### 2.4.1. Bidirectional GRU Backend

The bidirectional GRU backend is introduced by Zhou et al. [2]. It uses two bidirectional Gated Recurrent Unit layers with a hidden size of 512 in each direction, enabling the model to capture temporal dependencies flowing both forward and backward across the feature frames. This design is well-suited for the smooth and continuous temporal patterns found in EMG activity.

#### 2.4.2. Transformer Backend

In this study, we modified the Transformer backend originally proposed by Zhou et al. [2]. A standard positional encoding is added before entering the Transformer, supplying the model with information about temporal order [30]. The representation is then processed by a 3-layer Transformer encoder, where each layer contains four attention heads and a 512-dimensional feed-forward module. Unlike the GRU, the Transformer does not rely on recurrence, as it uses self-attention, allowing every time step to directly attend to all others. This allows the model to capture long-range and non-linear temporal interactions more effectively and in parallel. For the GRU backend, frame-wise logits are produced across the temporal dimension, whereas for the Transformer backend, the resulting sequence representation is collapsed into a fixed-size vector via global average pooling over time before being passed to the same final classification layer that predicts the 101 classes.

#### 2.4.3. Residual Mamba (ReMamba) Backend

The ReMamba backend applies a unidirectional selective state-space Mamba block to the temporal feature sequence produced by the convolutional frontend [31]. The implementation uses a model width of 256, 24 latent states, a convolution width of 4, and an expansion factor of 2, with a residual path connecting the block input and output. The output sequence is averaged over time and passed to the classifier. This pooling strategy matches that used for the Transformer and provides a common sentence-level output for non-recurrent models.

#### 2.4.4. BiMamba Backend

BiMamba is introduced here as a bidirectional Mamba-inspired backend [31] that derives context from earlier and later frames [32,33]. Unlike ReMamba, the BiMamba blocks do not use the selective state-space scan; instead, each block combines a gated linear projection with a depth-wise temporal convolution and a residual connection. In this work, the temporal token sequence produced by the CNN frontend is first normalized using Layer Normalization, followed by a lightweight temporal convolutional mixing stage and a linear projection into the BiMamba model dimension before temporal modeling. The temporal module is built from paired forward and backward blocks at each layer. For the backward path, the input sequence is first reversed, processed, and then restored to its original order. The outputs from both directions are then combined through summation and passed through a final normalization step. The training protocol is described in Section 2.4.5.

#### 2.4.5. Training and Evaluation Protocol

All models were trained from scratch using the Adam optimizer, cross-entropy loss, an initial learning rate of 0.0003 and a batch size of 32, for 50 epochs. The learning rate was kept constant and then halved every five epochs, starting after epoch 20 with a weight decay of 0.0005 for the MFSC GRU, Transformer, and ReMamba models, and after epoch 12 with a weight decay of 0.001 for the remaining models. The model obtained after the final epoch was evaluated once on the test subjects, while the validation subjects were used only to monitor training. Validation accuracy had stabilized before epoch 50 for all models. Each model was trained once with a fixed random seed. For the GRU and BiMamba backends, the token-level logits are averaged before the loss, whereas the Transformer and ReMamba backends pool the tokens before the classifier. Each recording is classified into one of 101 fixed sentence classes (100 voiced daily/medical sentences plus one blank class), and performance is reported as top-1 sentence-level classification accuracy. Because test evaluations are conducted on 20 unseen subjects reading the same 101 fixed sentence classes as the training cohort, performance measures subject-independent generalization across speakers rather than generalization to unseen sentences or unconstrained speech. All experiments were run with PyTorch 2.4.1 on an NVIDIA GeForce GTX 1650 Ti GPU (NVIDIA Corporation, Santa Clara, CA, USA).

### 2.5. Analysis of Temporal Variation and Speaker-Invariance 

To examine possible reasons for the better generalization of the WST-based pipeline to unseen participants, we quantified how much of the feature variance is related to the speaker rather than to the sentence. The stored features of the 20 test subjects (19,806 recordings) were used before any model or adaptation. Each recording was represented by its time-averaged feature vector (216 values for both MFSC and WST), and each feature was z-scored across recordings. The variance was then divided into the proportions explained by the speaker and by the sentence class (η^2^).

At the participant level, sentence templates were computed from the other 19 subjects (leave-one-speaker-out). For each held-out subject, we measured the distance of their sentence means from these templates, normalized by the spread of the templates, and the proportion of sentences closest to the correct template. MFSC and WST were compared across the 20 subjects using the two-sided Wilcoxon signed-rank test.

Temporal variation was measured on the same stored features. Each feature array was arranged as a sequence of frames along its time axis, with *D* = 216 values per frame for both representations. Since the frame spacing differs between the representations (50 ms for MFSC and approximately 33 ms for WST), the change was computed between frames 100 ms apart (two MFSC frames and three WST frames) as the L2 norm of the difference divided by $\sqrt{D}$. The values were averaged per recording and per subject and compared across the 20 subjects using the two-sided Wilcoxon signed-rank test.

## 3. Results and Discussion

The following experiments benchmark MFSC and WST with each temporal backend defined in Section 2.4: GRU, Transformer, ReMamba, and BiMamba. Test-set recognition, computational demand, and inference behavior are considered jointly, so that the two frontends are assessed under the same modeling protocol.

### 3.1. Benchmarking MFSC vs. WST Across Temporal Backends

This subsection compares MFSC and WST test results for the four backends trained as described in Section 2.4.5. Table 1 lists test accuracy together with trainable parameters, floating-point operations (FLOPs), and measured latency for GRU, Transformer, ReMamba, and BiMamba.

#### 3.1.1. Test Accuracy

Table 1 shows test accuracy across the backend models along with trainable parameters, floating-point operations (FLOPs), and measured latency. Across all temporal backends, the WST pipeline consistently achieves higher test accuracy compared to the MFSC pipeline, with accuracies ranging from 85.01% to 85.82% and 73.46% to 79.18%, respectively. This corresponds to an improvement of approximately 6 to 12 percentage points in favor of WST. For the WST representation, the Transformer model delivered the highest subject-independent performance, reaching a sentence-level accuracy of 85.82% on the 19,806 test recordings of the 20 unseen subjects. In contrast, MFSC achieved its best result with the ReMamba model, achieving a sentence-level accuracy of 79.18%. The smallest performance gap between WST and MFSC features occurs with the ReMamba backend, where WST achieves 85.01% compared to MFSC’s 79.18%, while the largest disparity is observed with the BiMamba model, where WST reached 85.45% compared to 73.46% for MFSC. Compared with the baseline results reported by Zhou et al. [2], both proposed feature representations achieved higher accuracy with the GRU backend; accuracy increased from 75.53% to 85.31%, while with the Transformer backend, accuracy increased from 63.54% to 85.82%.

#### 3.1.2. Model Parameters

As shown in Table 1, changing the frontend does not alter the trainable parameter count within a given backend: GRU contains 9.52 M parameters, Transformer 3.94 M, ReMamba 2.81 M, and BiMamba 7.36 M, including the 30 adapter parameters. The accuracy differences therefore cannot be attributed to additional learned capacity.

#### 3.1.3. FLOPs

The WST models exhibit a moderate increase in FLOPs compared to MFSC because the WST input contains more frames (53 versus 36), which enlarges the feature maps of the convolutional frontend (e.g., an increase from 0.22 to 0.31 G for GRU and Transformer, an increase from 0.22 to 0.35 G for ReMamba, and an increase from 0.83 to 1.23 G for BiMamba); see Table 1. This higher computational cost aligns with Chen et al. [34], who demonstrated that input representations with larger temporal dimensions increase the required floating-point operations (FLOPs) when processed by identical backbone networks.

#### 3.1.4. Model Latency

Latency was measured for a single recording (batch size 1) as the mean of 100 forward passes after 20 warm-up passes on an NVIDIA GeForce GTX 1650 Ti GPU, and the input adapter of the BiMamba models is included. Feature extraction is not included in the latency calculation, as the MFSC and WST features were precomputed offline, and the measurement is intended to compare the models themselves. Although both GRU and BiMamba exploit context in two temporal directions, Table 1 shows lower latency for GRU. This difference is better explained by their computational pathways than by parameter count. GRU operations have long benefited from highly tuned GPU kernels in major learning frameworks, including cuDNN optimizations for recurrent networks [35]. Mamba, by comparison, constructs sequence states using selective, scan-based state-space operations [31]. Our bidirectional implementation executes distinct forward and reverse blocks and then fuses their outputs, which adds sequential work and memory traffic relative to a one-way Mamba. The consistently longer BiMamba latency observed for both frontends accords with these implementation differences. These measurements should nevertheless be interpreted as specific to the evaluated code and hardware. The same consideration explains why a WST configuration can incur slightly more FLOPs yet complete inference faster: realised latency also depends on kernel efficiency, parallel execution, and memory-access behavior.

#### 3.1.5. Subject-Wise Test Accuracy

**Figure 4:**
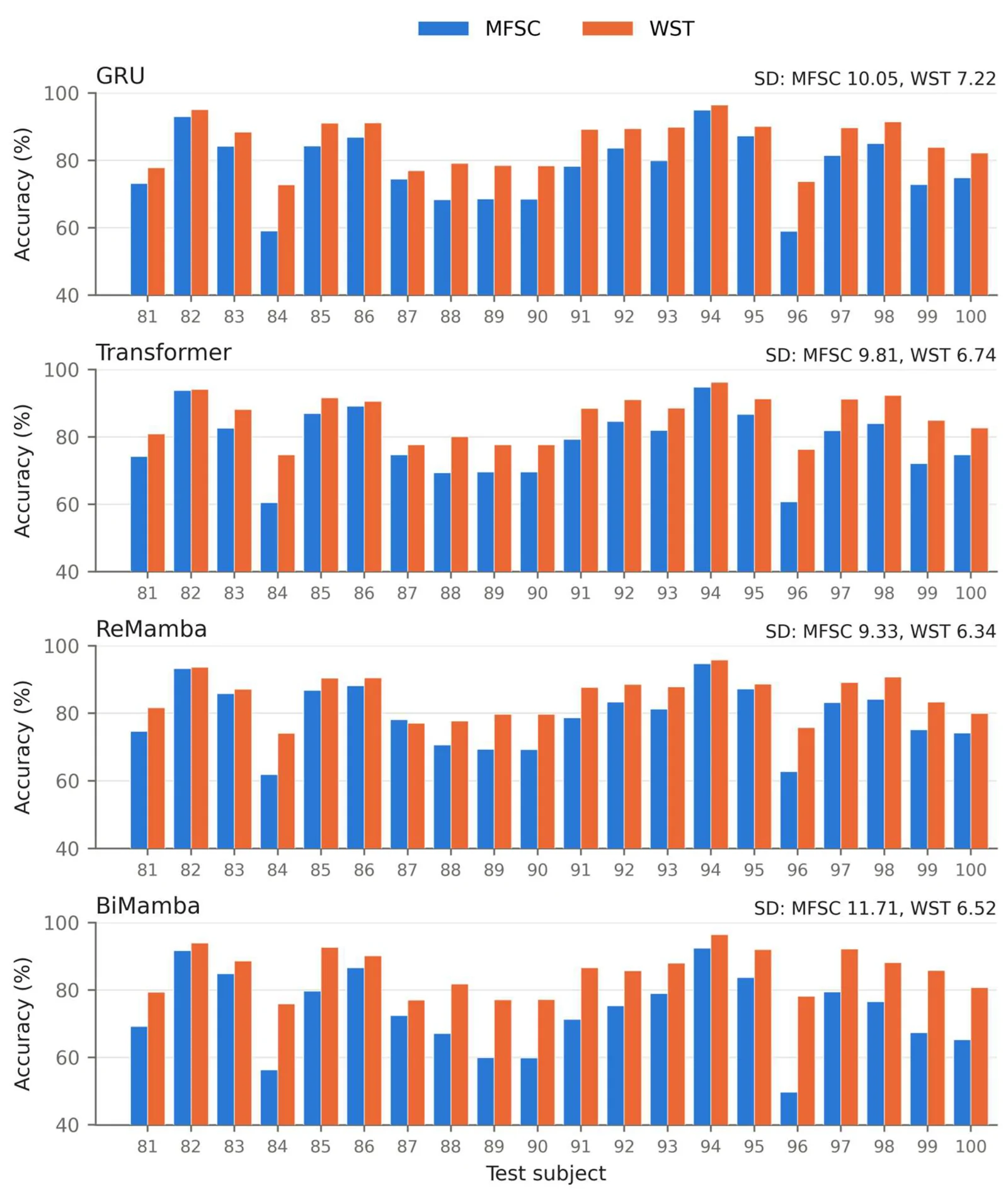
Subject-wise test accuracy (%) across MFSC and WST representations with four temporal backends (20 unseen test subjects).

We next examine whether each frontend-backend pairing behaves consistently across individuals, rather than obtaining its aggregate gain from only a few strong participants. Figure 4 reports accuracy separately for the 20 held-out subjects. Since recordings from the same subject are not independent, statistical testing was performed at the subject level using the paired accuracy differences (WST—MFSC), their mean with a 95% confidence interval (CI) computed from the 20 paired differences using the t-distribution with 19 degrees of freedom, and the two-sided Wilcoxon signed-rank test. WST improved the mean accuracy by 7.4 (95% CI 5.6–9.2), 7.3 (5.3–9.2), 5.9 (4.0–7.8) and 12.0 (8.9–15.1) percentage points for GRU, Transformer, ReMamba and BiMamba, respectively, with 20, 20, 19 and 20 of the 20 subjects improving (*p* ≤ 1.2 × 10^−4^). As each model was trained once with a fixed seed, these intervals reflect variability across subjects and not across training runs. Across the four MFSC models, the subject-wise standard deviation ranges from 9.33 to 11.71, whereas WST narrows this range to 6.34–7.22. The advantage is especially pronounced for Subjects 84 and 96, the most difficult cases under MFSC. Subject 84 obtains 56.34–61.84% with MFSC but 72.73–75.90% with WST. For Subject 96, the corresponding intervals are 49.75–62.69% and 73.72–78.13%. Thus, scattering improves not only participants who are already recognized accurately but also those for whom the conventional representation performs poorly. The reduced dispersion and gains on these difficult cases suggest lower sensitivity to inter-subject variability and more stable performance on people absent from training.

Because the improvements recur across participants and sequence models, they point to an advantage of the WST pipeline itself. Comparable subject-level analyses remain uncommon in EMG speech research, where cohorts are often small. Wu et al. [36], for instance, obtained 92.7% accuracy for an 11-word, neck-EMG task involving two speakers. Gaddy and Klein [9] reported a word error rate of 42.2% for open-vocabulary recognition from one speaker. Scheck et al. [37] studied cross-speaker EMG-to-speech conversion with four speakers recorded over multiple sessions and showed benefits from more diverse training observations. Chen et al. [38] similarly used multi-speaker self-training to improve voiced EMG-to-speech conversion beyond a 23.3% WER baseline. In comparison, our participant-disjoint test covers 20 previously unseen speakers. Gains are present throughout almost the entire cohort and reach approximately 28 percentage points for the hardest individuals, supporting pipeline-level generalization at a substantially larger subject scale.

While the mathematical properties of the WST offer theoretical invariance to small signal deformations, physical robustness against external disturbances, such as explicit acoustic/electrical noise perturbations, electrode displacement, or cross-session temporal shifts, was not directly tested in this study and remains an essential target for future evaluation.

#### 3.1.6. Class-Wise Test Accuracy

Table 2 summarizes class-level accuracy obtained from the MFSC and WST confusion matrices for every backend. Across models, scattering improves both average performance and its balance over the vocabulary. Mean class accuracy rises from 0.7797 to 0.8534 for GRU, 0.7862 to 0.8586 for Transformer, 0.7920 to 0.8504 for ReMamba, and 0.7349 to 0.8548 for BiMamba, equivalent to absolute gains of roughly 0.06–0.12. Class-wise dispersion also decreases, consistent with the subject-level pattern in Figure 4. Standard deviation falls from 0.1182 to 0.1001 for GRU, 0.1110 to 0.0987 for Transformer, 0.1085 to 0.0957 for ReMamba, and 0.1137 to 0.0871 for BiMamba. The minimum accuracy is higher with WST in every comparison: 0.4091 versus 0.5505 for GRU, 0.4646 versus 0.5578 for Transformer, 0.4242 versus 0.4949 for ReMamba, and 0.3889 versus 0.6061 for BiMamba. Hence, much of the benefit comes from rescuing weak classes rather than further improving those already near the ceiling. Indeed, maximum accuracy remains between 0.9898 and 1.0000 for both frontends. The number of classes attaining at least 0.85 provides another view of the same effect. Depending on the backend, only 13–37 MFSC classes reach this threshold, compared with 56–62 WST classes. This broad expansion of high-accuracy coverage shows that the improvement is neither confined to selected sentences nor dependent on one temporal architecture. Instead, WST increases discriminability across the vocabulary and yields a more even class-performance profile.

### 3.2. Comparison with State-of-the-Art Studies

Table 3 compares the present study with studies in the literature on the same dataset or architecture. The closest published comparison is Zhou et al. [2], who evaluated the same AVE Speech task using the same participant-disjoint split. Their EMG-only BiGRU and Transformer results were 75.53% and 63.54%, respectively, compared with 85.31% and 85.82% for the present WST-based pipeline. Within this study, the corresponding MFSC pipelines achieved 77.94% and 78.59%, providing more direct pipeline comparisons. Related results require additional context: Wu et al. [16] used MFSC features and a parallel inception CNN, but their 90.76% result used randomly partitioned pooled recordings rather than participant-disjoint evaluation. Gaddy and Klein [9] used convolutional features and a Transformer for single-speaker EMG-to-speech synthesis, with performance measured by transcription WER rather than sentence-classification accuracy. Satterlee et al. [14] evaluated four sentences from one participant and incorporated language-model processing. Ghezaiel et al. [28] provide a precedent for combining scattering with a CNN, although their acoustic speaker-identification task differs from EMG speech recognition. These studies therefore offer dataset or methodological context rather than a common accuracy ranking. The present findings support improved accuracy of the evaluated WST-based pipeline on unseen AVE Speech participants; they do not isolate the effect of the transform or establish inherent speaker invariance.

### 3.3. Temporal Variation and Speaker-Invariance Results

Table 4 summarizes the results for the speaker-invariance analysis. Speaker identity explained 69.0% of the MFSC feature variance but only 12.1% of the WST feature variance, whereas the sentence class explained 2.3% and 6.2%, respectively. All 20 held-out subjects were closer to the other speakers’ sentence templates with WST (*p* = 1.9 × 10^−6^), and the correct template was found more often (8.7% versus 2.8%; 19 of 20 subjects; *p* = 1.6 × 10^−4^). Thus, the WST-based features appear to carry less speaker-specific information, which is consistent with their higher accuracy on unseen subjects. Part of this effect is likely related to the normalization steps of the WST pipeline, since applying a similar per-speaker normalization to both feature sets gave comparable sentence-to-speaker ratios (0.19 for MFSC and 0.20 for WST).

Table 4 also reports the temporal variation in both representations. After normalization by the feature dimensionality and matching of the time interval, WST did not show lower temporal variation than MFSC; the change over 100 ms was higher with WST for all 20 subjects (0.315 versus 0.279; *p* = 1.9 × 10^−6^). This is consistent with the short averaging scale (16 samples) and the Δ/Δ^2^ features of the WST pipeline, which retain faster temporal changes than the 200 ms MFSC windows. Hence, the accuracy advantage of the WST-based pipeline is not explained by smoother temporal trajectories, but it is consistent with its lower speaker dependence.

### 3.4. Exploratory Effect of MFSC-Style Filtering on WST

At the start of this analysis, MFSC-style preprocessing was applied to the WST features to assess their effect. The data split and training settings were the same as for the WST baseline; however, this run used the frontend pooling of the ReMamba models and a separately initialized channel projection, so the result should be regarded as exploratory. Applying MFSC-style preprocessing to WST results in a decrease in test accuracy of 2.16 percentage points compared to the unfiltered WST representation; see Table 5. This suggests that the MFSC filtering does not benefit WST, although the decrease in performance cannot be attributed to filtering alone.

### 3.5. Clinical and Practical Implications

EMG-based speech recognition provides a reliable communication approach when audible speech is either unavailable or undesirable [3,7]. The need for such an approach is increasing, as the proportion of elderly individuals continues to grow and many of them face difficulties in articulating their needs due to hearing or speech impairment [2]. Audio-based ASR is not suitable in these situations, since its accuracy diminishes significantly in high-noise environments or when acoustic signals cannot be reliably captured [2], and it cannot be used by laryngectomy patients who are no longer able to produce sound after the removal of the vocal folds [3]. Visual speech recognition (lip-reading) avoids the acoustic problem, yet it is sensitive to lighting conditions and has difficulty in distinguishing between words with similar mouth shapes [2]. On the other hand, surface EMG captures the activity of the articulatory muscles directly; therefore, it is not affected by acoustic noise or lighting, is well suited to laryngectomy patients whose vocal tract remains almost intact [3], and can be integrated into wearable systems [11,14,15]. Nevertheless, EMG signals are susceptible to variability due to physiological differences among users and electrode shifts [2], and speaker-dependent systems require large amounts of training data from each user, which is not normally available in practice [3]. The need for per-person and session-to-session calibration is therefore considered a key technical hurdle, and sEMG decoding models that generalize across people have only recently been demonstrated for gesture and handwriting tasks [24]. Accordingly, the focus of the present study is not only to maximize accuracy, but also to identify the EMG representation that transfers best to new speakers who did not contribute any labeled training data.

In practice, the lower speaker dependence of the WST-based pipeline suggests that a recognizer trained on a group of speakers could be used by a new speaker without first collecting labeled recordings from that speaker, which reduces the need for user-specific training and calibration. This is particularly important for users who cannot provide large amounts of training data [3]. For such a user, the best WST model (Transformer, 85.82%) would correctly recognize approximately six out of seven sentences, whereas the MFSC model using the same Transformer backend (78.59%) would correctly recognize approximately four out of five sentences. In relative terms, WST reduces the sentence error rate from 21.41% to 14.18%, corresponding to an approximately 33.8% reduction in recognition errors. Because this improvement was observed in 79 of the 80 participant–backend pairs, the performance gain is consistent across almost all evaluated speakers rather than being driven by a small number of favorable outliers.

Moreover, the sentences of the AVE Speech corpus include medical requirements, e.g., “I keep coughing” and “My blood pressure is high” [2]. Hence, the evaluated task corresponds to a phrase-based communication aid that could support patients in communicating with caregivers, assist elderly individuals in expressing daily needs, and enable private communication or communication in noisy environments [2,3]. From a practical perspective, the combination of improved speaker-independent accuracy and wearable-compatible EMG acquisition provides a basis for developing communication interfaces that require less user-specific training. These applications remain prospective and require validation in clinical populations and under realistic conditions.

## 4. Conclusions

This study presented a systematic investigation of EMG feature representation for subject-independent speech recognition, comparing Mel-Frequency Spectral Coefficients (MFSC) and the Wavelet Scattering Transform (WST) across four temporal backend architectures: GRU, Transformer, ReMamba, and BiMamba. All models were evaluated on a closed-set classification task over 101 fixed sentence classes (100 daily/medical Mandarin phrases plus one blank class) recorded during voiced (vocalized) speech across 20 unseen test speakers. Across all backends, WST consistently outperformed MFSC, achieving a peak top-1 accuracy of 85.82% with the Transformer backend.

Participant-level variance analysis revealed that speaker identity explained less feature variance in WST than in MFSC (12.1% vs. 69.0%), consistent with greater resilience of the WST-based pipeline to inter-speaker variability. After controlling for feature dimensionality and frame spacing, WST did not exhibit lower temporal variation than MFSC, indicating that its performance gain is not explained by trajectory smoothness but is consistent with its lower speaker dependence. Furthermore, applying conventional bandpass and notch filtering prior to WST reduced performance by 2.16 percentage points, suggesting that such filtering may suppress discriminative EMG signal components.

While these results indicate that the WST-based pipeline provides a less speaker-dependent representation for fixed-phrase voiced EMG classification, we emphasize that physical robustness to external perturbations—such as electrode displacement, additive noise, and cross-session signal degradation—was not directly evaluated and remains an essential target for future testing. Future work will focus on expanding this framework to real-time execution, open-vocabulary continuous decoding, clinical populations, multi-seed statistical evaluations, additional matched controls/ablation studies, and explicit feature-pipeline component ablations.

## Figures and Tables

**Table 1:** Benchmarking of Mel-Frequency Spectral Coefficients (MFSC) and a Wavelet Scattering Trans-form (WST) feature representations across temporal architectures. Green and orange shading highlights the best and worst values in each column among the current models, respectively. For the parameters FLOPs and latency, the lowest value is the best.

	Representation	Temporal Model	Accuracy (%)	Params (M)	FLOPs (G)	Latency (ms)
Current models	MFSC	GRU	77.94	9.52	0.22	2.28
		Transformer	78.59	3.94	0.22	2.43
		ReMamba	79.18	2.81	0.22	2.02
		BiMamba	73.46	7.36	0.83	4.77
	WST	GRU	85.31	9.52	0.31	1.81
		Transformer	85.82	3.94	0.31	2.35
		ReMamba	85.01	2.81	0.35	2.41
		BiMamba	85.45	7.36	1.23	4.77
Zhou et al. [2]	MFSC	GRU	75.53			
		Transformer	63.54			

**Table 2:** Class-wise accuracy statistics derived from confusion matrices for MFSC and WST across all models. Green and orange shading highlights the best and worst values in each column, respectively. For Std, the lowest value is the best.

Representation-Model	Mean	Std	Min	Max	≥0.85
MFSC-GRU	0.7797	0.1182	0.4091	0.9898	32
MFSC-Transformer	0.7862	0.1110	0.4646	0.9898	37
MFSC-ReMamba	0.7920	0.1085	0.4242	1.0000	33
MFSC-BiMamba	0.7349	0.1137	0.3889	0.9847	13
WST-GRU	0.8534	0.1001	0.5505	0.9950	59
WST-Transformer	0.8586	0.0987	0.5578	1.0000	62
WST-ReMamba	0.8504	0.0957	0.4949	0.9949	56
WST-BiMamba	0.8548	0.0871	0.6061	0.9898	58

**Table 3:** Comparison with published studies using the same dataset or related features and neural architectures. Published results are taken from the cited studies and were not re-evaluated here. Differences in tasks, protocols, modalities, and metrics preclude a common performance ranking. WER denotes word error rate.

Study	Dataset and Task	Features and Architecture	Evaluation Protocol	Reported Result
Zhou et al. (2025) [2]	AVE Speech; 100 voiced Mandarin sentences plus blank; 100 participants	MFSC; BiGRU and Transformer	Participant-disjoint: 70 train/10 validation/20 test	Accuracy: 75.53% BiGRU; 63.54% Transformer
Present study	AVE Speech; same 101-class task; 100 participants	WST-based pipeline; GRU and Transformer	Participant-disjoint: 70 train/10 validation/20 test	Accuracy: 85.31% GRU; 85.82% Transformer (for 100 subjects, 10 sessions for each subject, each session 101 classes)
Wu et al. (2022) [16]	Separate Mandarin EMG corpus; 100 phrases plus empty class; 28 participants	MFSC; parallel inception CNN	Random 80/20 split of pooled recordings for the reported result	Accuracy: 90.76% (for 28 subjects)
Gaddy and Klein (2021) [9]	Single-speaker English corpus; open-vocabulary EMG-to-speech synthesis	Learned convolutional features; Transformer; WaveNet vocoder	Held-out utterances from one speaker	WER: 42.2% from automatic transcription of synthesized speech
Satterlee et al. (2025) [14]	Separate corpus; four sentences; one participant	Few-shot Siamese network; moving windows; trigram language model	Within-participant evaluation	Sentence accuracy after language modeling: 93.33% EMG; 95.25% EMG + EEG
Ghezaiel et al. (2020) [28]	TIMIT and LibriSpeech audio; closed-set speaker identification	Wavelet scattering and CNN	Acoustic speaker identification rather than EMG sentence recognition	Methodological comparison only; no comparable EMG sentence metric

**Table 4:** Temporal variation and speaker dependence of MFSC and WST features for the 20 test subjects. Best values are highlighted in green, and worse values are highlighted in orange.

Measure	MFSC	WST	WST Better in (*p*)
Temporal variation over 100 ms (L2/$\sqrt{D}$)	0.279	0.315	0/20 (1.9 × 10^−6^)
Variance explained by speaker (η^2^)	0.690	0.121	—
Variance explained by sentence (η^2^)	0.023	0.062	—
Distance to other speakers’ templates	32.7	4.25	20/20 (1.9 × 10^−6^)
Correct template match (%)	2.8	8.7	19/20 (1.6 × 10^−4^)

**Table 5:** Exploratory effect of MFSC-style preprocessing on WST performance.

Representation	Temporal Model	Accuracy (%)
WST (baseline)	Transformer	85.82
WST + MFSC-style preprocessing	Transformer	83.66

## Data Availability

The AVE Speech dataset used in this study is publicly available at https://huggingface.co/datasets/MML-Group/AVE-Speech (accessed on 15 October 2025). The developed code forms part of an ongoing research project and is available from the authors upon reasonable request.
